# Simulation of hypoxia of myocardial cells in microfluidic systems

**DOI:** 10.1038/s41598-020-72660-w

**Published:** 2020-09-23

**Authors:** Anna Kobuszewska, Elżbieta Jastrzębska, Kamil Żukowski, Zbigniew Brzózka

**Affiliations:** 1 Chair of Medical Biotechnology, Faculty of Chemistry, Warsaw University of Technology, Warsaw, Poland; 2 CEZAMAT, Warsaw University of Technology, Warsaw, Poland

**Keywords:** Lab-on-a-chip, Disease model

## Abstract

The paper presents a newly designed microfluidic system that allows simulation of myocardial hypoxia by biochemical method. The geometry of the microsystem was designed in such a way, that quantitative fluorescent measurements using a spectrofluorometric plate reader was possible. Biochemical simulation of hypoxia was carried out using potent mitochondrial oxidative phosphorylation uncoupler—Carbonyl cyanide-4-(trifluoromethoxy)phenylhydrazone (FCCP). Two cardiac cell lines were used in the study—rat cardiomyoblasts (H9C2) and human cardiomyocytes. The effectiveness of biochemical simulation of hypoxia was studied using two fluorescent dyes: carbocyanine iodide (JC-1) and Fluo-4. Changes in the mitochondrial membrane potential and concentration of intracellular calcium ions were tested. The major novelty of this research was the applying the microfluidic system to create hypoxia conditions for cardiac cells using the biochemical approach. In further studies, the presented hypoxia model could be used to develop new methods of treatment of ischemic heart disease for example in cell therapy based on stem cells.

## Introduction

Heart diseases are the major cause of death worldwide. Annually, 17.3 million people die because of this type of ailments, which accounts for 31% of all deaths in the world. It is estimated that by 2030, the number of deaths would increase to 23.6 million. Ischemic heart disease (IHD) is the main cause of mortality of people suffering from heart diseases^1^. IHD is a condition resulting from chronic insufficient supply of cardiac cells with oxygen and nutrients. Oxygen is an essential factor for the proper functioning of cardiac cells. Oxygen deficiency (hypoxia) could interfere with the life processes of cells such as oxidative mechanisms of respiration, metabolic processes using glucose or fatty acids, cell proliferation, gene expression or ATP synthesis. Chronic hypoxia leads to myocardial necrosis and irreversible cell damages (loss of heart muscle), which results in heart failure^2,3^. Currently, the most effective treatment method of heart failure is a heart transplantation. However, the main limitations of this method are the number of donors and the possibility of the transplant rejection^4^. Therefore, it is necessary to develop an alternative treatment method.

Microfluidic systems (called *Lab-on-a-Chip*) could be useful tools in the development of the cellular model of IHD. These microsystems have a lot of advantages such as: the possibility of designing geometry and dimensions mimicking the natural microenvironment of the cells, control of cell culture conditions, physical parameters similar to those occurring in physiological conditions (surface-to-volume ratio (SAV), an effective culture volume (ECV), laminar flow), the possibility of conducting real-time analysis and they are an alternative tool to animal studies^5^. Microsystems also enable the creation of a model of a myocardial tissue (called *Heart-on-a-Chip* systems) in a form of monolayer^6,7^ or a three-dimensional cell culture^8,9^. Additionally, to the advantages described above, there are features of the microsystems specific for heart cell culture. To create a heart model in microscale, it is necessary to know and to mimic the properties of this specific tissue. Because heart is characterized by both parallel cardiac muscle fiber and complex electrochemical dynamics, these factors are mimicked in *Heart-on-a-chip* systems^10,11^. For this purpose dynamic (perfusion) conditions and additionally nanofibers, microgrooves are used to obtain parallel orientation of the cells^7,11^. Cell stretching, is the next feature, which can be successfully simulated in the microfluidic devices. It can be obtained thanks to the usage of a thin membrane and changing the pressure. Controllable stimulation with electrical field is the next feature, which can be used in microscale to obtain parallel orientation of the cells as well as their contraction^9,11^. *Heart-on-a-Chip* systems, characterized by these properties, could be used to study the physiology of heart cells under in vitro conditions and to evaluate the cytotoxicity of drugs, used to treat heart diseases or other ailments^8,10,11^. For several years, researchers have also tended to develop models of diseases, including IHD. For this purpose, it is necessary to create hypoxic conditions in the microsystems.

In the literature, several methods of generating hypoxia used in in vitro studies were described. Hypoxic chambers are commonly used due to the possibility of controlled gas supply. These reactors allow for the generation of preferred conditions by mixing oxygen, carbon dioxide and nitrogen^12^. Khanal et al. investigated the human prostate cancer cells (PC3) response to an anticancer drug under normoxia and hypoxia conditions (1% O_2_)^13^. In turn, Yang et al. used the hypoxic chamber to evaluate how hypoxia conditions (2.5% O_2_) influence on three-dimensional neural stem cell (NCS) culture^14^. However, achievement of an equilibrium between the oxygen pressure in a culture medium and an atmosphere in hypoxia chamber takes several hours. Moreover, at the time of removing the cells from the hypoxia chamber for testing, oxygen from the atmosphere begins to diffuse into the medium, which changes the culture conditions. Another widely used solution to simulate hypoxia in a microsystem are the gas channels. These channels enable controllable supply of a gas to the microsystem. In the microsystems, the gas supply channel is separated from the culture chamber by a thin membrane made of poly(dimethylsiloxane) (PDMS). Rexius-Hall et al. *designed a microsystem that contained two gas supply *channels. The use of two gases (oxygen and nitrogen) allowed for creation of an oxygen gradient that diffused through the thin PDMS membrane^15^. The gas channels were also used in the studies on the migration of cells under hypoxic condition. A polycarbonate film was used to separate the gas channels from the culture chambers, because of the low oxygen diffusion coefficient^16^. An interesting solution used in bioreactors or microfluidic systems is the use of scavengers. The scavengers are chemical substances which decrease an oxygen tension in a culture media and allows for evaluating many conditions of the cell culture in one device. Wang et al. used Na_2_SO_3_ as a scavenger to generate an oxygen gradients under various conditions in the microsystem^17^. The creation of conditions with different oxygen concentrations enable to study the effect of combined therapy (antitumor drug with hypoxia) on cancer cells. In turn, Takano et al. presented the microsystem, which contained a water jacket with the dissolved ascorbate (oxygen scavenger). By using the water jacket, it was possible to reduce the oxygen content in the culture medium to 5% and the cell culture under hypoxic conditions^18^. The biochemical method could be also used to simulate hypoxia under in vitro conditions. For this purpose, substances such as rotenone^19^, carbonyl cyanide-4-(trifluoromethoxy)phenylhydrazone (FCCP)^20^, desferrioxamine^21^ or cobalt(II) chloride^22^ are used. The advantage of using this type of substance is simplicity and fast simulation of hypoxia (tens of minutes). Thanks to this, the biochemical method is commonly used in the microsystems^22,23^. However, they might have a negative effect on cell viability, metabolism or morphology.

Because ischemic heart disease is the main cause of death worldwide, it became necessary to develop new methods of treatment. We presented a microfluidic system for simulating of myocardial hypoxia. In contrast to other microsystems presented in the literature, our device is characterized by simple geometry. In addition, the presented microsystem was designed to be compatible with a multi-well plate reader, which allowed for quantitative analysis. The major novelty of this research is the applying the microfluidic system to create hypoxia conditions for cardiac cells using the biochemical approach. Moreover, it was proved that the proposed approach allowing achieving hypoxia conditions faster and simpler in comparison with more common solutions (e.g. hypoxia chambers). The degree of hypoxia was evaluated by studying changes in the membrane potential of mitochondria and intracellular calcium ion concentration on the two cardiac cells lines. To the best of our knowledge, it is the first time when hypoxia was stimulated on human cardiomyocytes under microfluidic conditions. Moreover, the obtained results showed that microfluidic conditions influence cell response to the hypoxia to compare with macroscale study. Also, the assessment of changes in intracellular calcium ions concentration under influence of hypoxia obtained by biochemical way was evaluated for the first time.

## Results and discussion

### Design and fabrication of the microsystem

**Figure 1:**
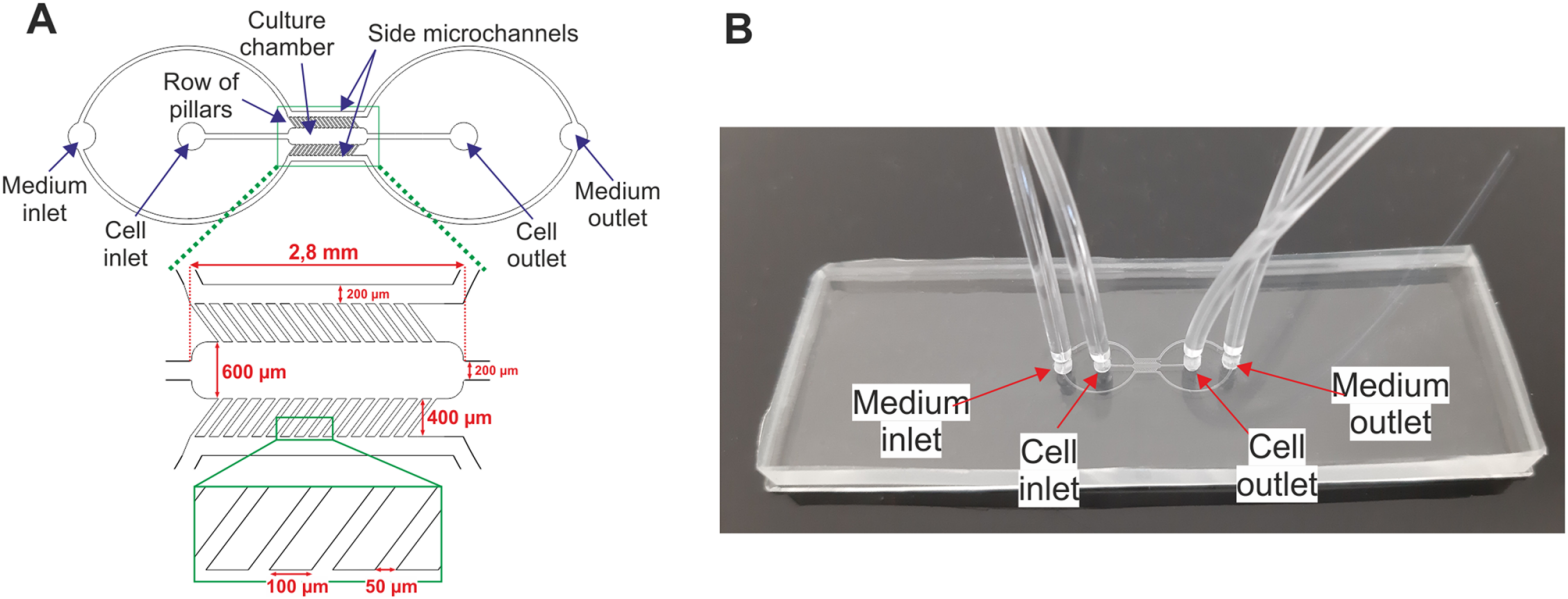
(**A**) A geometry of the designed microsystem with the dimensions of the microstructures. (**B**) The fabricated microsystem.

**Figure 2:**
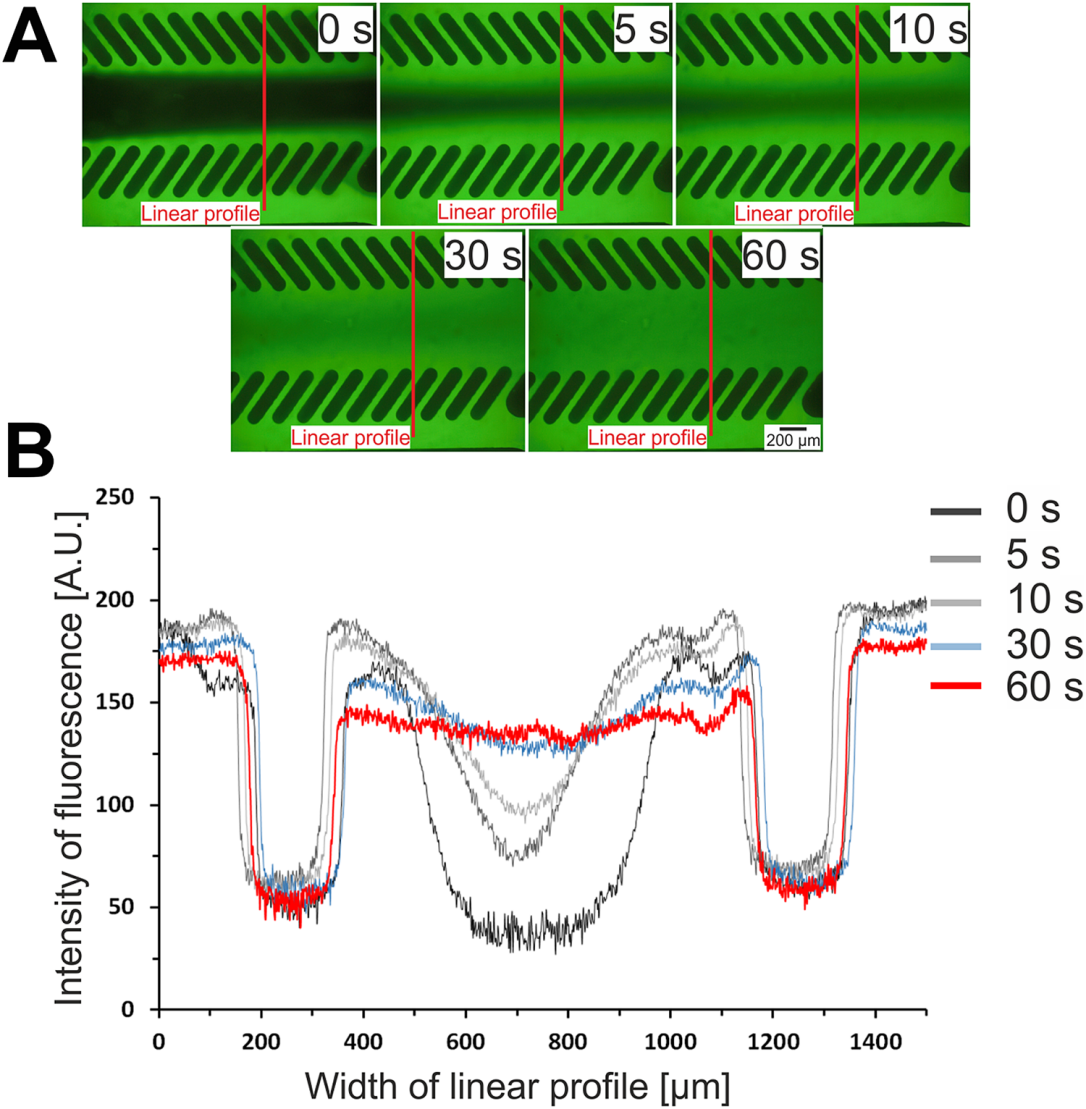
(**A**) A visualization of the diffusion of culture medium from the side microchannels into the culture microchamber using fluorescein solution. (**B**) Linear profile of changes in intensity of fluorescence in the culture microchamber.

The designed microsystem is consisted of two layers. The bottom layer is a glass slide. The top layer, made of PDMS layer with the microchannels network was fabricated using replica moulding technique. The poly(methyl methacrylate) (PMMA) stamp for the fabrication of the top PDMS layer was made using micromilling technique. The presented microsystem consists of the main microchannel that is used to culture the cardiac cells. On both sides of the main microchannel there are two side microchannels that are used to provide the culture medium and other solutions. The main microchannel and the side microchannels are separated by rows of longitudinal micropillars. Microchannels between the micropillars imitate endothelial barriers. These barriers separate the cardiac cells from perfusion medium and protect from shear forces^24^. At the same time, microchannels between the micropillars enable to provide nutrients to the cells. Moreover, the dimensions of the main microchannel were selected to correspond to the diameter of one well in the 384-well plate. This allowed for quantitative measurements on the plate reader using special designed adapter (see Supplementary Materials). The geometry of the designed microsystem and the fabricated microsystem are presented in Fig. 1. Visualization of the flow of the culture medium with the fluorescein solution was performed. Based on the obtained pictures (Fig. 2A), it was found that the fluorescein solution diffuses between the pillars into the culture microchamber. The Fig. 2B shows changes in the linear profile of fluorescence intensity for different times (0, 5, 10, 30 and 60 s) from the moment of introducing fluorescein solution into the microsystem. It was observed that the fluorescein solution evenly filled the culture microchamber in 60 s.

### Evaluation of hypoxia treatment in macroscale

**Figure 3:**
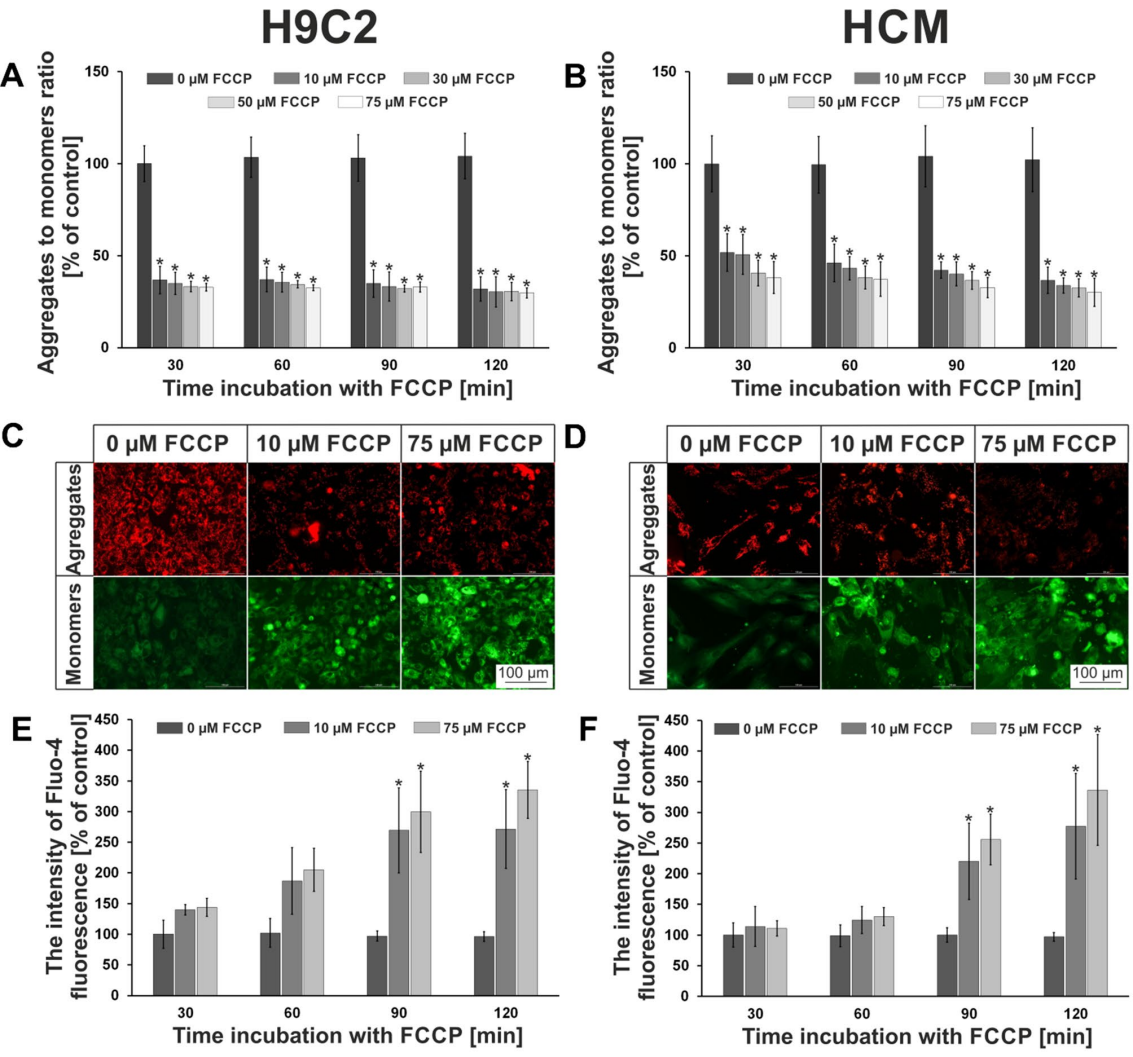
The macroscale results of hypoxia optimization. The results obtained in tests on changes in mitochondrial membrane potential for (**A**) H9C2 and (**B**) HCM cell lines. (**C**) H9C2 cells and (**D**) HCM cells after 120 min incubation with FCCP. Both cell lines were stained with JC-1. The results obtained in tests on changes in concentration of intracellular calcium ions for (**E**) H9C2 and (**F**) HCM cell lines. N ≥ 3, asterisks indicate *p* < 0.05.

In our research, stimulation of myocardial hypoxia was induced using biochemical pathway. The FCCP compound was used for this purpose. FCCP is a potent mitochondrial oxidative phosphorylation uncoupler. FCCP induces translocation of H^+^ ions across inner mitochondrial membrane, thereby blocking ATP synthesis^25,26^. The use of FCCP allows to create conditions very similar to those prevailing under in vivo conditions^27^. To optimize cardiac cell (H9C2 or HCM) cultures under hypoxia conditions different concentrations of FCCP (10, 30, 50, 75 µM) and exposure times (30, 60, 90, 120 min) were tested. Hypoxic effect of FCCP was determined by cationic carbocyanine dye—JC-1. For accurate quantitative assessment of the hypoxic effect of FCCP on cardiac cells, the fluorescent intensity ratios of the two forms of JC-1 (aggregates/monomers) were compared. The obtained results showed that FCCP application to cardiac cells caused significantly decreased of aggregates to monomers ratio. For H9C2 cells, the decrease above 50% after 30 min of incubation with the lowest concentration with FCCP was observed (Fig. 3A). There were no significant differences between other concentrations and incubation times with FCCP. The ratios of both forms of the dye are maintained at a similar level. In case of HCM cells, after 30 min of incubations with FCCP for concentrations of 10 and 30 µM, the decrease of less than 50% was noticed (Fig. 3B). However, for two other concentrations, the aggregates to monomers ratio decreased above 50%. After 60 min of exposure from FCCP, the aggregates to monomers ratio decreased to above 50% for all tested concentrations. Similar to the H9C2 cells, the ratio of both forms of JC-1 maintained at the same level up to 120 min of incubation. Microscopic observations were also carried out. It was found that the intensity of red fluorescence (corresponding to aggregates; live cells) decreased with increasing concentration of FCCP, whereas the intensity of green fluorescence (corresponding to monomers; apoptotic cells) increased. Images of the cells stained with JC-1 and incubated with FCCP for 120 min are presented in Fig. 3C (H9C2 cells) and Fig. 3D (HCM cells). The obtained results indicated that 30 min for H9C2 cells or 60 min for HCM cells and 10 µM concentration of FCCP are optimal conditions for simulating hypoxia of myocardial cells. In vivo, cardiomyocytes start to die after 20 min of hypoxia^28^. Thus, the results obtained for the H9C2 line are more similar to the conditions prevailing during hypoxia in the human body. This proved that we have managed to create the model of hypoxia of the cardiac cells in a macroscale. Mathur’s group also used JC-1 to determine changes of the mitochondrial membrane potential^29^. However, they study potential changes in primary cultures of neonatal rat cardiomyocytes using carbonyl cyanide *m*-chlorophenylhydrazone (mClCCP). They determined how conditions cause the formation aggregates and monomers of JC-1 using a flow cytometry. In contrast to this work, we performed study in micro- and macro-scale using other cell lines and simulation agent. In the literature, there are also examples of research in which hypoxia is simulated using hypoxic chambers^30,31^. However, the hypoxia simulation process in the hypoxic chamber is long. It varies from 18 to 48 h. The advantages of the biochemical method we used is the rapid time after which the cells are introduced into the stage of apoptosis. The use of JC-1 dye allowed us to confirm that FCCP causes apoptosis of cardiac cells.

One of the phenomena occurring in cells during their apoptosis is the increased amount of calcium ions in the cytosol. Ca^2+^ ions play a key role in the transmission of cellular information and signals between the cells^32^. Moreover, the calcium ions are especially important in cardiac cells, because they are responsible for the generation of contraction. During our test, it was checked whether the biochemical simulation of hypoxia in cardiac cells also causes an increase in the intracellular concentration of calcium ions. To the best of our knowledge, this type of research has not been carried out yet on H9C2 and HCM cells. For this purpose, the Fluo-4 dye was used. Fluo-4 binds with calcium ions, which increases the emission of green fluorescence (λ_ex_ = 494 nm, λ_em_ = 506 nm)^33^. It was tested whether the biochemical simulation of hypoxia caused the increase in intracellular concentration of calcium ions for H9C2 cells (Fig. 3E). The amount of Ca^2+^ ions increased with the time of cell incubation with FCCP. After 30 min of the hypoxia simulation, the concentration of calcium ions was 1.5 higher compared to the control for both tested FCCP concentrations (10 and 75 µM). After 60 min of incubation a twofold increase in the amount of calcium ions was observed, and after 90 min triple increase. There were no differences between 10 and 75 µM of FCCP. After 120 min of incubation with FCCP, content of intracellular calcium ion remained unchanged for 10 µM, while for 75 µM it increased to 350%. For the HCM cell line, similar results were obtained (Fig. 3F). For the first two exposure times, a slight increase in the calcium ion concentrations was noticed—to approximately 150%. Also, there were no significant differences between both tested concentrations. A significant increase (to approximately 250%) in the content of calcium ions in the cells was observed after 90 min of incubation. After 120 min, the concentration of calcium ions in HCM cells increased to approximately 280% for samples incubated with 10 µM FCCP solution. For 75 µM of FCCP the increase of calcium ions equaled approximately 350%. Our test proved that biochemical simulation of hypoxia caused the increase of intracellular calcium ions in cardiac cells. As mentioned earlier, the increasing of concentration of calcium ions in the cells is one of apoptosis symptom. Thus, the observed Ca^2+^ increase in H9C2 and HCM cells may indicate that the cardiac cells have entered into a state of apoptosis. Similar results were obtained by the *Zhang’s* group, however another method of simulation of hypoxia was used. Conditions for hypoxia was obtained using hypoxia incubator (95% N_2_ and 5% CO_2_). They evaluated how hypoxia/reoxygenation (H/R) injury affects cellular processes. It was proved that H/R induced increase of calcium ions in cardiac microvascular endothelial cells, which led to cell apoptosis^34^. We proved that biochemical method of hypoxia simulation is as effective as using a hypoxic chamber. In turn, Fernández-Morales and Morad also tested whether acute hypoxia has influence on intracellular calcium ions concentration changes in cardiac cells^35^. However, in this research another method of hypoxia stimulation was used. Hypoxia solution (bubble with 100% N_2_; PO_2_ < 5 mmHg) was used to achieve rapid changes in the extracellular oxygen content. In contrast to our studies, it turned out that acute hypoxia caused decrease of intracellular calcium ions.

### Evaluation of hypoxia treatment in microscale

**Figure 4:**
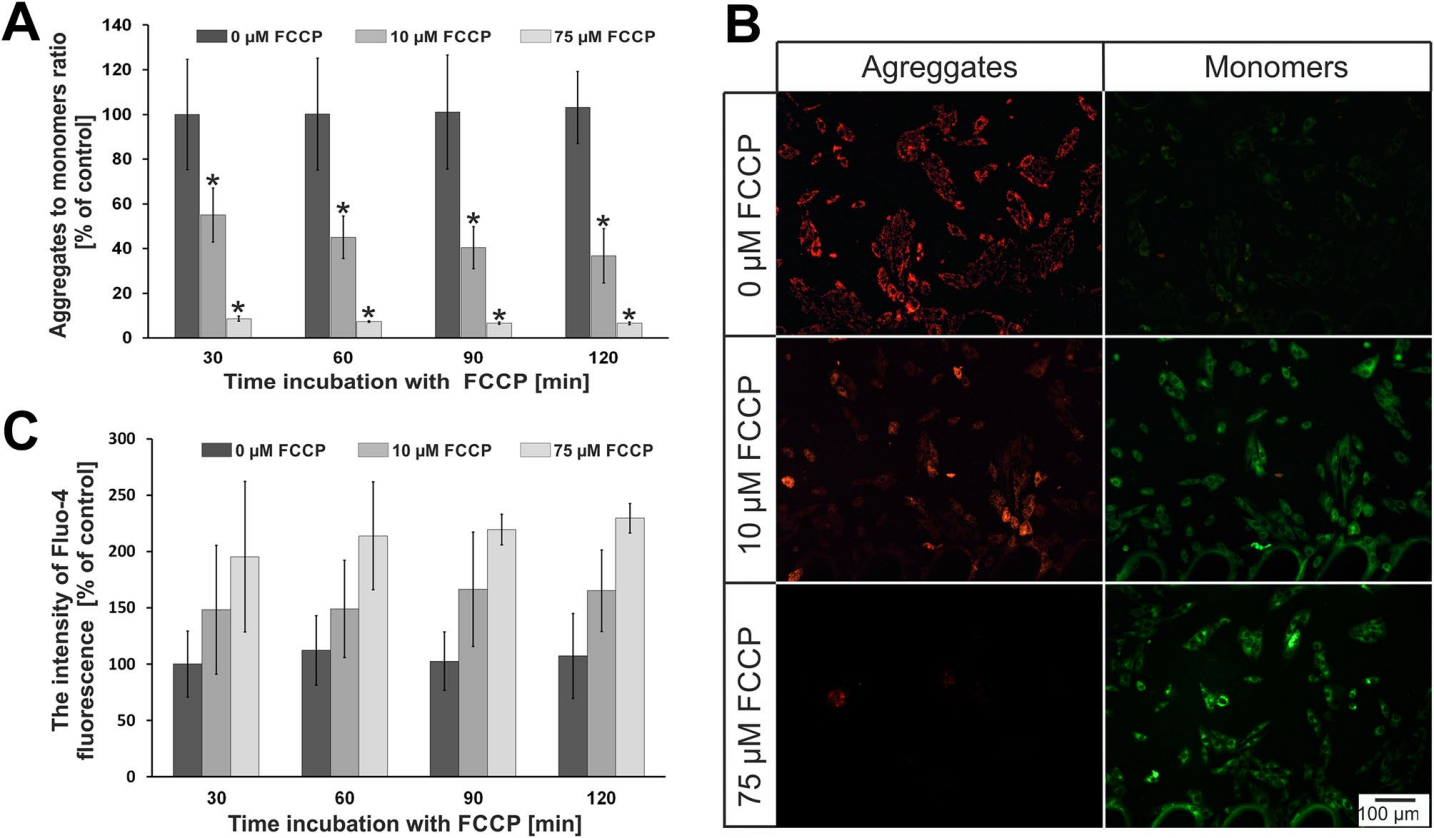
(**A**) The results of changes in mitochondrial membrane potential for H9C2 cells. N ≥ 3, asterisks indicate *p* < 0.05. (**B**) H9C2 cells after 120 min incubation with FCCP. The cells were stained with JC-1. (**C**) The results of changes in concentration of intracellular calcium ions for H9C2 cells.

**Figure 5:**
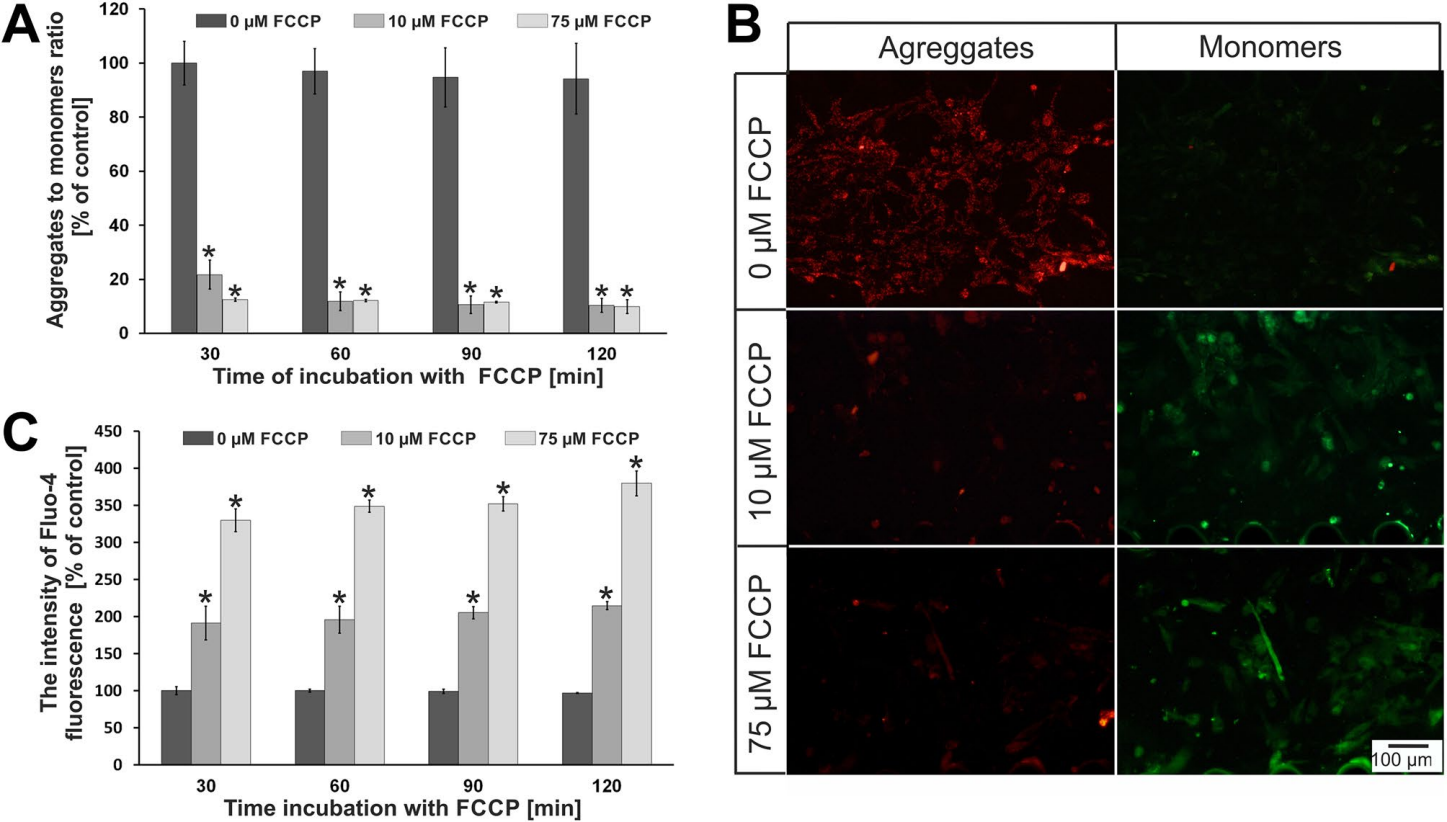
(**A**) The results of changes in mitochondrial membrane potential for HCM cells. N ≥ 3, asterisks indicate *p* < 0.05. (**B**) HCM cells after 120 min incubation with FCCP. The cells were stained with JC-1. (**C**) The results of changes in concentration of intracellular calcium ions for HCM cells. N ≥ 3, asterisks indicate *p* < 0.05.

Based on the research in macroscale, hypoxia simulation was performed in microscale. For this purpose, the microsystems were applied. As we described in the introduction, microfluidic conditions better mimic a conditions prevailing in the human body than static conditions (multi-well plates)^36^. Based on macroscale analysis, we decided to investigate two concentrations of FCCP in the microsystems (10 µM and 75 µM). These two concentrations were selected to assess whether the increase in FCCP concentration affects the state of cell culture in the microscale, which was not observed in the macroscale. It could answer that culture conditions are important for cardiac cell research. Four times of incubation were tested (30, 60, 90, 120 min). We wanted to select the optimal conditions for simulating hypoxia in the microsystem. The results of our tests are presented in Fig. 4 (H9C2 cells) and Fig. 5 (HCM cells). In the case of rat cardiomyoblasts, the effect of FCCP concentration on the changes in the mitochondrial membrane potential was observed (Fig. 4A). The JC-1 aggregates to JC-1 monomers ratio decreased with increasing of FCCP concentration. For cell incubated with 10 µM FCCP a decrease of the ratio to approximately 55% was observed after 30 min of incubation. The ratio of the both dye forms slightly decreased (to 40%) after 120 min of incubation. However, for a concentration of 75 µM the decrease of aggregates to monomers ratio (to 10%) was observed only after 30 min. This level was maintained at the same level for 120 min of incubation. At the same time, microscopic observation was carried out. The obtained results for the incubation time of 120 min are shown in Fig. 4B. Similarly to tests conducted in the macroscale, it was found that the intensity of red fluorescence decreased with increasing concentration of FCCP, whereas the intensity of green fluorescence increased. The results obtained during microscopic observations confirmed the results of the quantitative analysis. Similarly to macroscale, studies on the effect of hypoxia on the intracellular concentration of calcium ions were also conducted (Fig. 4C). It was found that calcium ions concentration increased 1.5-fold for 10 µM FCCP and approx. twofold for 75 µM FCCP after 30 min of incubation. The amount of concentration of calcium ions maintained at the same level for both tested concentration of FCCP up to 120 min.

For human cardiomyocytes (HCM line), the effect of FCCP on changes in the mitochondrial membrane potential were also observed (Fig. 5A). The JC-1 aggregates to JC-1 monomers ratio decreased by about 80% for cells incubated with 10 µM FCCP after 30 min. In the following minutes, the ratio decreased to approx. 10% and remained at this level up to 120 min. In turn, the ratio decreased to approx. 10% for the concentration of 75 µM FCCP and maintained at the same level until the end of the incubation. The differences of the results between HCM and H9C2 cell lines were noticed. For H9C2 cells, values of the aggregates to monomers ratio were from 40 to 55% for 10 µM FCCP. In turn for HCM line, these values decreased to 10–20% for the same concentration. This indicated that human cardiac cells were more sensitive to FCCP than rat cardiac cells. As in case of the H9C2 line, microscopic observations were carried out, which confirmed the results obtained from quantitative measurements (Fig. 5B). A decrease of intensity of the red fluorescence was observed for the increase of FCCP concentration. At the same time, the intensity of the green fluorescence increased with increasing FCCP concentration. Additionally, a significant increase of calcium ions content inside the cells was observed for the HCM line for the tested FCCP concentrations (Fig. 5C). It equaled approx. twofold for 10 µM FCCP and approx. 3.5-fold for 75 µM. The content of calcium ions in the cells was maintained at the same level during the entire incubation time. Similar results were noticed in macroscale studies. However, it was found that the increase of intracellular calcium ion concentration for HCM line was higher than for H9C2 line.

We created models of myocardial ischemia on two cardiac cell lines: rat cardiomyoblasts (H9C2) and human cardiomyocytes (HCM). H9C2 cell line was chosen because it is a model line to imitate myocardial tissue. However, the rat organism is different from human. In addition, H9C2 cells are precursor cells to cardiomyocytes and do not exhibit all the characteristics of cardiomyocytes. For these reasons human cardiomyocytes were also investigated. Based on the presented results, it was found that the model of myocardial ischemia was successfully obtained in the microsystem. The presented model was created in the designed microsystem using the biochemical method using FCCP to simulate hypoxia. We proved that our method is effective and universal for various cell lines. Our conclusions were confirmed by two independent tests. A decrease in mitochondrial membrane potential is an evidence of mitochondrial dysfunction and the initiation of the apoptotic process. The research with JC-1 dye were performed in macro- and micro-scale. The obtained results for the cells cultured on multi-well plates and in the microsystems were different for two tested cell lines. For H9C2, the JC-1 aggregates to JC-1 monomers ratio decreased to approx. 50% for the tests performed in macroscale. These results were not both FCCP concentration and incubation time dependent. However, these factors had influence on the JC-1 aggregates to JC-1 monomers ratio in the tests carried out in the microsystem. For 10 µM FCCP, the aggregates to monomers ratio was 50% after 30 min and decreased to 40% after 120 min. A higher decrease of the aggregates to monomers ratio was observed for 75 µM FCCP (approx. 15%) than for 10 µM FCCP. Similar results were obtained for HCM cell line. For macroscale, the aggregates to monomers ratio was approx. 50% and no changes were noticed with the increase both FCCP concentration and incubation time. For microscale, the aggregates to monomers ratio in HCM cells equaled about 10% for each measuring point (except 10 µM FCCP after 30 min). Differences between macro- and microscale may be caused by cell culture conditions. The cells are cultured under static conditions on the multi-well plates. The microenvironment prevailing in microsystems is characterized by a laminar flow and high ratio of surface to volume (SAV). The low value of an effective culture volume (ECV) indicates that the cells better control their microenvironment under microfluidic conditions. In addition, the scale of the microstructures corresponds with the cellular microenvironment in vivo. These factors affect cell culture and because of them microfluidic conditions better reflects in vivo conditions than static conditions (multi-well plate)^5,37–39^. However, a flow of reagents could induce shear stress and it could have a negative effect on cell viability. Differences of the results between the tested cell lines, obtained in the microsystem, could be caused by higher sensitivity of HCM than H9C2 cells to external factors (a flow rate, FCCP). Apoptosis was also demonstrated by the increase in intracellular concentration of calcium ions, which was evaluated using Fluo-4 dye. Hypoxia in the microsystem was simulated after 30 min, which is similar time to the one in which myocardial necrosis occurs in in vitro conditions. It was noticed that 90 min and 120 min incubation with FCCP caused the highest value of calcium ion concentrations in both cell lines in macroscale. Contrary, the same value of calcium ion concentrations was obtained in the microfluidic systems for each tested incubation time. These differences may result from various cell culture conditions (static and perfusion). It was also observed that there was higher concentration of calcium ions in HCM than H9C2 cells. This may be due to the fact that cardiomyocytes need more calcium ions in cytosol for the proper functioning of the contractile apparatus. Moreover, the number of apoptotic HCM cells in the microsystem was higher than H9C2 cells. This could be caused by higher sensitivity of the cardiomyocytes to FCCP action. The experiment time was significantly shortened by using the proposed biochemical method. Thanks to that, our method of hypoxia simulation has the advantage over the traditional hypoxia chamber^14^ or gas channel^16^, in which the state of hypoxia was obtained after several hours or days. To the best of our knowledge, there are not many examples in the literature of hypoxic myocardium model in the microfluidic systems. Khanal et al.^40^ presented the microsystem, which contained an additional microchannel with a fluorescent dye sensitive to oxygen level. In order to obtain the hypoxic condition, the microsystem was placed in a polycarbonate box that was supplied with nitrogen. The cardiac cells were incubated for 3 h under hypoxic conditions. In contrast to this work, we managed to reduce simulation time to 30 min using biochemical method of hypoxia simulation. This proved the advantage of the method used by us. Biochemical method of hypoxia is often used in the studies. Rotenone was used as a factor, which induced hypoxia in cardiomyocytes^19^. It was observed that rotenone causes expression of pre-preondothelin-1 in cardiac cells, which leads to mitochondrial dysfunction, apoptosis and mimic hypoxia. In other studies, FCCP was used and its effect on the reactive oxygen species (ROS) formation and glutathione (GSH) secretion in lung cancer cells were investigated^20^. The results showed that FCCP increased the content of ROS and GSH in cells, leading to apoptosis. However, these studies were carried out in macroscale on lung cells. They used a different cellular model than proposed in our research. Biochemical reagents simulating hypoxia were also used in the microsystems. Chen et al. studied the effect of hypoxia on the angiogenesis of cell co-culture [human retinal pigmental epithelial cells (APRE-19) and human umbilical vein endothelial cells—(HUVEC)]^22^. CoCl_2_ was used to simulate hypoxic conditions in the designed microsystem and caused directional growth of HUVEC and cell death of APRE-19. In the literature, there are also examples of microfluidic systems in which other methods of hypoxia simulation were used. Wang et al. used hypoxia in the studies of the cytotoxic effect of tirapazamine and cisplatin on lungs cells (A549)^23^. The tests were carried out in the designed microfluidic system containing a gas channel separated from a culture microchamber by a PDMS membrane. NaOH and pyrogallol were introduced into the gas channel. This solution allowed to obtain a gradient of oxygen content in the culture microchamber. It turned out, that hypoxic conditions indicated higher cytotoxicity of the tested drugs. The effect of hypoxia on the differentiation of human neural stem cells (hNSC) were also investigated^14^. The research was carried out on 3D cell culture in a hydrogel in the microfluidic system. The hypoxia was simulated by using incubator supplied with nitrogen. Based on the research, it was found that hypoxia did not cause stem cells differentiation. In these studies, various method of hypoxia has been used as an anti-cancer agent or a factor affecting the behavior of various cells (mostly cancerous). In addition, the sign of apoptosis such as changes in the mitochondrial membrane potential and the concentration of intracellular calcium ions were not evaluated in above mentioned studies. For these reasons, it is difficult to compare our research to the literature reports. The aim of our research was to create a cellular model of hypoxic myocardial, which in subsequent tests may be used for research on the regeneration of the damaged tissue. To the best of our knowledge, there are no examples in the literature of cellular model of IHD in the microfluidic systems. Because of the limitation in the contemporary treatment of ischemic heart disease, it is important to create model that mimic in vivo conditions. We believed that the presented model could be used to better understand IHD at the cellular level, which in turn will contribute for the development of new therapies.

## Conclusions

Optimization of the stimulation of myocardial ischemia on two cardiac cell lines was performed in the macroscale. The research was carried out using rat cardiomyoblasts (H9C2 cell line) and human cardiomyocytes (HCM cell line). Hypoxia was simulated by biochemical method using potent mitochondrial oxidative phosphorylation uncoupler—FCCP. The condition of the cell culture was evaluated by two spectrophotometric methods. In the first of them, JC-1 was used to study changes in the mitochondrial membrane potential. The decrease of ratio of the both dye forms indicates the cell apoptosis. Changes in the intracellular calcium ions concentration was also tested using Fluo-4. Based on the obtained results, it was found that hypoxic myocardial model could be obtained during 30 min using biochemical method.

In this paper, we also demonstrated the microfluidic system, which was used to simulate the hypoxia of cardiac cells. It was proved that under the influence of biochemical hypoxia simulation, the cells entered into the state of apoptosis. This is evidenced by the decrease in the mitochondrial membrane potential and the increase in the concentration of calcium ions inside the cells. We also managed to prove that the biochemical method of inducing hypoxia in the microfluidic systems is as effective as traditional hypoxic chamber. Thanks to the use of microchannels networks and rows of micropillars that imitate the endothelial barrier, the microsystem allows for creation of conditions similar to the conditions in the human heart. The microfluidic systems with the obtained cell cultures could be used as a model of the hypoxic myocardial. In the further stages of work, such model could be used to develop new methods of IHD treatment or to study the regeneration of the myocardium using stem cells.

## Materials and methods

### Design and fabrication of microsystem

The microsystem structure was designed with CAD program (AutoCad, Autodesk). The geometry of the presented microsystem consists of two inlets and two outlets connected with microchannels (Fig. 1A). Cell inlet was connected with the main culture microchamber (2.8 mm long, 600 µm wide and 200 µm high) and was used to introduce the cell suspension into the microsystem. The medium inlet was connected with two side microchannels (200 µm wide and 200 µm high) and was used to provide culture medium. The main microchannel and side microchannels were separated by two rows of micropillars (10 µm long, 400 µm wide, 200 µm high, gap 50 µm).

The designed microsystem was composed of two layers: bottom, a microscopic glass slide (Chemland) and top, poly(dimethylsiloxane) (PDMS) (Sylgard 184, DowCorning) with the microstructures. The microstructures were fabricated using micromilling and replica moulding techniques. The micromilling technique was used for poly(methyl methacrylate) (PMMA) master fabrication, according the method described previously^9^. The pre-polymer was mixed with the curing agent in the weight ratio of 10:1 to prepare the PDMS mixture. The PDMS mixture was degassed and then poured over the PMMA structure and cured at 70 °C for 1 h. Holes in the PDMS layer were drilled. The PDMS layer with the microstructures was tightly bonded with a microscopic slide glass using oxygen plasma treatment. The dimension and arrangement of the culture microchamber fit within one well of 384-well plate, which enabled for quantitative fluorescent measurements using a spectrofluorometric plate reader. The measurements were performed using a designed adapter that was fitted to the plate reader (see Supplementary Materials). A 5 × 10^–4^ µM fluorescein solution was used to visualize the flows in the microsystem. The fluorescein solution was introduced into the side microchannels and a distilled water into the culture microchamber at the same time using peristaltic pump (a flow rate). Then, the flow was switched off and the time of fluorescein diffusion to the culture microchamber between micropillars was measured. Changes in linear profile of the fluorescence intensity in the culture microchamber in different times was also investigated using cellSens image analysis software (Olympus).

### Cell cultures

Two different cardiac cell lines: rat cardiomyoblast (H9C2 cell line, ECACC, Sigma-Aldrich) derived from embryonic myocardium and human cardiomyocytes (HCM cell line, ScienCell) derived from ventricles of adult heart were used in the experiments. H9C2 cells were maintained in Dulbecco’s Modified Eagle Medium (DMEM, Sigma-Aldrich) supplemented with 10% v/v fetal bovine serum (FBS, Gibco), 1% v/v 100 mM penicillin–streptomycin (Sigma-Aldrich) and 1% v/v 25 mM l-glutamine (Sigma-Aldrich). HCM cells were maintained in Dulbecco’s Modified Eagle Medium: Nutrient Mixture F-12 (DMEM/F12, Gibco) supplemented with 10% v/v fetal bovine serum (FBS, Gibco), 1% v/v 100 mM penicillin–streptomycin (Sigma-Aldrich), 1% v/v 25 mM l-glutamine (Sigma-Aldrich), 1% v/v 100 mM sodium pyruvate (Sigma-Aldrich), 0.01% v/v cardiac myocyte growth supplement (CMGS, ScienCell) and 0.01% v/v MEM non-essential amino acids (NEAA, Sigma-Aldrich). The cells (H9C2 and HCM) were cultured in a humidified incubator (37 °C, 5% CO_2_, HERA-cell 150, Thermo Scientific). For the preparation of cell suspensions, monolayer cell cultures were washed with phosphate buffered saline (PBS, Sigma-Aldrich) and trypsinized with 0.25% Trypsin (Sigma-Aldrich). Then the cells were resuspended in culture medium.

### Cell cultures and hypoxia treatment in the macroscale

H9C2 or HCM cell suspensions (density of 10^4^ cells per well) were seeded in 96-well plates and allowed to attach overnight. Potent mitochondrial oxidative phosphorylation uncoupler—carbonyl cyanide-4-(trifluoromethoxy)phenylhydrazone (FCCP) (Abcam) was used to optimize cardiac cell culture under hypoxia conditions in macroscale. 24 h after seeding, FCCP was added to cell culture for a final concentration of 10–75 µM and incubated in 37 °C and 5% CO_2_ for 30, 60, 90 and 120 min. For tests with H9C2 cells, FCCP solutions were prepared in DMEM without phenol red (Sigma-Aldrich) supplemented with 10% v/v FBS, 1% v/v 100 mM penicillin–streptomycin and 1% v/v 25 mM l-glutamine. While for tests with HCM cells, FCCP solutions were prepared in DMEM without phenol red supplemented with 10% v/v FBS, 1% v/v 100 mM penicillin–streptomycin and 1% v/v 25 mM l-glutamine, 1% v/v 100 mM sodium pyruvate, 0.01% v/v cardiac myocyte growth supplement and 0.01% v/v MEM non-essential amino acids.

### Cell cultures and hypoxia treatment in the microscale

Before the experiments, the fabricated microsystems were sterilized using ultraviolet (UV) light (exposure for 30 min) and 70% v/v ethanol. Then, the microsystems were filled with the culture medium and placed in an incubator for 24 h to provide appropriate culture conditions. Then, H9C2 or HCM cell suspensions (density of 10^6^ cells/ml) were introduced into the microsystem by designed inlet. Flow rates of introduced suspension was 10 µl/min. To enable cell adhesion to the glass substrate, the microsystem was placed in an incubator at 37 °C and 5% CO_2_ for 24 h (HERA-cell 150 m Thermo Scientific). FCCP solution was introduced into the culture microchamber by side channels (a flow rate of 1 µl/min, 10 min) for a final concentration of 10 or 75 µM and incubated in 37 °C and 5% CO_2_ for 30, 60, 90 and 120 min. For tests with H9C2 and HCM cells, FCCP solutions were prepared analogously to the macroscale. The FCCP solution was exchanged for PBS before each measurement (a flow rate of 1 µl/min, 10 min). The FCCP solution was introduced again into the microsystem (a flow rate of 1 µl/min, 10 min) and incubated for the next 30 min.

### Cell observation and analysis

Observations of cell cultures was carried out using an inverted fluorescence microscope coupled with a charge-coupled device camera (CCD camera) (Olympus IX71). For data acquisition and analysis, cellSens image analysis software (Olympus) was used. Mitochondrial membrane potential, concentration of cellular calcium ions were analyzed to evaluate the hypoxic injury dynamic of cardiac cells. Cationic carbocyanine dye—5,5′,6,6′-tetrachloro-1,1′,3,3′-tetraethylbenzimidazolyl carbocyanine iodide (JC-1) (Biotium) was used to determine changes of mitochondrial membrane potential in cardiac cells caused by FCCP. JC-1 dye was used for detection of mitochondrial depolarization and membrane potential decrease, which is characteristic in early stage of apoptosis^41^. For cells cultures in macroscale, JC-1 dye was prepared with 5 µM final concentration and added to each well (100 µl) before hypoxia treatment. The cells were incubated with JC-1 in 37 °C and 5% CO_2_ for 30 min. After that time, dye was exchanged into a culture medium and the cells were incubated in 37 °C for next 60 min. 90 min is the time, which is needed to stabilize JC-1 in cardiac cells^41^. In case of cell cultures in microscale, 5 µM JC-1 solution was introduced into the microsystem by the side microchannels using peristaltic pump (a flow rate of 1 µl/min, 10 min) and incubated in 37 °C and 5% CO_2_ for 30 min. After that time, dye was exchanged into a culture medium (a flow rate of 1 µl/min, 10 min) and cell were incubated in 37 °C and 5% CO_2_ for next 60 min. The measurements (macro and microscale) were carried out in excitation wavelength of 475 nm and emission wavelength of 530 nm (monomers) or 590 nm (aggregates). The analysis of changes of intracellular calcium ions in cardiac cells was performed using fluorescent dye—Fluo-4. Fluo-4 dye was prepared with 4 µM concentration and added to each well (100 µl) before hypoxia treatment. The cells were incubated in 37 °C for 45 min. For cell cultures in microscale, 4 µM Fluo-4 solution was introduced into the microsystem by the side microchannels using a peristaltic pump (a flow rate of 1 µl/min, 10 min) and incubated in 37 °C and 5% CO_2_ for 45 min. The measurements (macro and microscale) were carried out at excitation wavelength of 494 nm and emission wavelength of 520 nm. In all tests, the intensity of fluorescence was measured using multiwall plate reader (Cytation 3, BioTek). The fluorescence intensity obtained for each tested time was calculated in relation to the fluorescent intensity obtained for the control in each test (0 µM FCCP, 30 min incubation). The experimental data were expressed as the mean ± standard deviation (SD) from at least three experiments. Statistical analysis was performed using one-way analysis of variance (ANOVA). *p* value of < 0.05 were considered statistically significant (asterisk indicates *p* < 0.05).

## Supplementary information

Supplementary Information.
