# Supplementary material for: Simulation of hypoxia of myocardial cells in microfluidic systems
Source: Sci Rep. 2020 Sep 23;10:15524. doi: 10.1038/s41598-020-72660-w (PMC7511941; doi:10.1038/s41598-020-72660-w)
Supplement: Supplementary file 1 — Supplementary Information. [file 41598_2020_72660_MOESM1_ESM.docx]

**SIMULATION OF HYPOXIA OF MYOCARDIAL CELLS IN MICROFLUIDIC SYSTEMS**

Anna Kobuszewska^1^, Elżbieta Jastrzębska^1^*, Kamil Żukowski^2^, Zbigniew Brzózka^1^

^1^Chair of Medical Biotechnology, Faculty of Chemistry, Warsaw University of Technology, Warsaw, Poland

^2^CEZAMAT, Warsaw University of Technology, Warsaw, Poland

*Corresponding author: Elżbieta Jastrzębska, ejastrzebska@ch.pw.edu.pl

**Supplementary Materials**

1. **Adapter for quantitative measurements in the microfluidic system**

A special adapter (Fig. S1.A) was fabricated to perform quantitative measurements in the microfluidic system. The adapter was made of polyether ether ketone (PEEK) using the micromilling method. The adapter dimensions (127 mm x 86 mm) correspond to those of a standard multi-well plate and thanks to that the adapter can be fitted into the plate reader. Two holes (25.4 mm x 76.2 mm) were performed in the adapter. The dimensions of the holes were the same as the dimensions of the developed microsystem. After placing the microsystem in the adapter, the arrangement of the culture microchamber corresponds to the size and the location of one well in a standard 384-well plate
(Fig. S1.B).


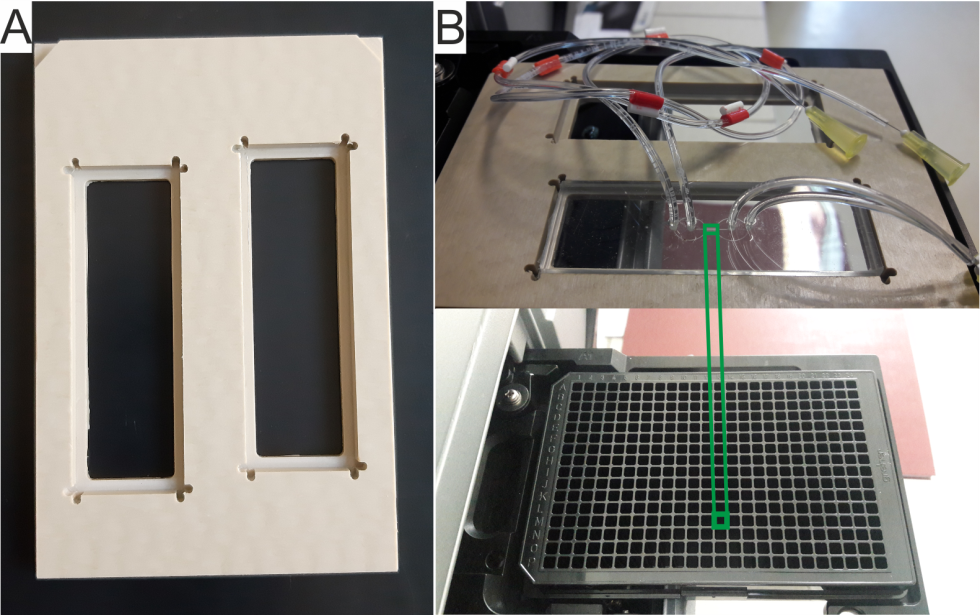


*Fig. S1. A) An image of the microsystem adapter for quantitative measurements. B) Image of the adapter with the microsystem in the plate reader (top). A standard 384-well plate is shown below to visualize the location of the culture microchamber.*
